# Improving Health and Cancer Services in Low-Resource Countries to Attain the Sustainable Development Goals Target 3.4 for Noncommunicable Diseases

**DOI:** 10.1200/JGO.18.00185

**Published:** 2018-11-30

**Authors:** Graeme W. Morgan, Kirsty Foster, Brendan Healy, Craig Opie, Vu Huynh

**Affiliations:** **Graeme W. Morgan**, **Kirsty Foster**, and **Vu Huynh**, The University of Sydney; **Craig Opie**, Royal North Shore Hospital, Sydney, New South Wales; and **Brendan Healy**, Icon Cancer Group, Brisbane, Queensland, Australia.

## Abstract

The United Nations Sustainable Development Goals 2015 to 2030 includes a specific goal for health (Sustainable Development Goal [SDG] 3) with 13 targets, including SDG3.4 for the control and treatment of noncommunicable diseases (NCDs), namely, cardiovascular diseases, cancer, diabetes, and chronic lung disease. There is considerable concern that SDG3.4 may not be achieved. The WHO Best Buys for NCDs has emphasized prevention, and although crucial, it alone will not achieve the 30% reduction in NCDs by 2030. Likewise, a strengthened health system is required as all NCDs are likely to require hospital facilities and community services for optimal management. This is a major problem for low-resource countries (LRCs) —that is, low-income countries and lower-middle-income countries—as most currently have a poorly developed health system, including cancer services, in need of upgrading. This is a result of the extreme poverty of LRCs, where 40% to 80% of the population live on less than USD $1.25 per day, with the average health spending by governments in low-income countries at $110 per person per year. In this article, we outline a comprehensive national cancer services plan for LRCs. Surgery, radiotherapy, and chemotherapy for cancer treatment also require input from other specialties, such as anesthesia, pathology, laboratory medicine, a blood bank, and diagnostic radiology. This will provide a focus for adding additional specialties, including cardiology, respiratory medicine, and psychiatry, to support the management of all NCDs and to contribute to the overall strengthening of the health system. The national cancer services plan for LRCs will require significant funding and input from both in-country and overseas experts in health, cancer, and finance working collaboratively. Success will depend on thoughtful strategic planning and providing the right balance of overseas support and guidance, but ensuring that there is in-country ownership and control of the program is essential.

## INTRODUCTION

In 1999, the WHO first recognized that noncommunicable diseases (NCDs), which consist of cardiovascular disease, cancer, diabetes, and chronic respiratory diseases, were a major health problem. By 2020, it was expected that NCDs would account for 73% of global deaths and 60% of the global burden of disease.^1^ In 2012, NCDs were responsible for approximately 38 million deaths per year or 68% of deaths worldwide. For premature deaths in 2012—between age 30 and 70 years—an estimated 52% were a result of NCDs.^2^ In 2015, a total of 47% of premature deaths that were attributable to NCDs occurred in low-resource countries (LRCs), namely, low-income countries (LICs) and lower-middle-income countries (Lo-MICs).^3^ These premature deaths primarily affect working persons who support a family so that preventing such deaths would have social and economic benefits for LRCs. NCDs have the common preventable risk factors of tobacco use, harmful use of alcohol, unhealthy diet, and physical inactivity, and the WHO has provided advice to assist member states to control these factors.^4-6^

The United Nations Sustainable Development Goals (SDGs) 2015 to 2030, or the 2030 Agenda, contains 17 goals and 169 targets. There is a specific goal for health (SDG3) that includes 13 targets, including SDG3.4: “To reduce by one third premature mortality from NCDs through prevention and treatment, and promote mental health and well-being.”^7^ In signing up to the 2030 Agenda, member states are asked to become involved in a range of goals and targets. Unfortunately, many member states, particularly in LRCs, are unlikely to be able to meet a number of goals and targets, including the SDG3.4 target of 30 by 30.^8,9^ This is a result of several factors, including a lack of government commitment or human and physical resources plus widespread poverty.

The WHO Best Buys—updated 2017—recommends interventions for NCDs based on Appendix 3 of the WHO Global Action Plan for NCDs 2013 to 2020.^10^ The majority of the recommendations are related to prevention rather than hospital care. Whereas prevention is an essential part of the management of NCDs, and even though it may be cost effective, it will take several decades for prevention measures, such as smoking cessation or human papillomavirus vaccination, to have any impact.

For control of cervical cancer, WHO Best Buys has recommended screening for women age 30 to 49 years using visual inspection and acetic acid, Papanicolau test, or human papillomavirus test linked with the timely treatment of precancerous lesions. No mention is made of invasive cervical cancer or the 2006 and 2014 WHO publications on the treatment of all stages of cervical cancer, including invasive cancer and not just precancerous lesions.^11,12^

It has recently been suggested that stage I and II invasive cervical cancer should be treated by either surgery or radiotherapy with or without chemotherapy.^3^ We would point out that all stages of invasive cervical cancer, including stage I and II, have been effectively managed using external beam radiotherapy and brachytherapy alone since at least the 1950s. However, both surgery or external beam radiotherapy and brachytherapy require a well-developed health system and support from cancer-related specialties.

The WHO Best Buys recommend that new cases of acute myocardial infarction be treated with aspirin, thrombolysis, or percutaneous coronary interventions. These would occur in a hospital with follow-up in primary health care facilities at a 95% coverage rate. Unless health care strengthening is prioritized in LRCs, the SDG Target 3.4 will not be attainable.

All NCDs are likely to require hospital facilities for the management of patients at some stage, particularly when complications develop. Establishing a sustainable health system is therefore essential if the 30 by 30 is to be achieved. In this article, we propose a national cancer services plan (NCSP) for LRCs. A comprehensive approach with surgery, radiotherapy, and chemotherapy, with involvement of anesthesia, medicine, pathology, diagnostic radiology, and palliative care, is essential for the diagnosis, treatment, and management of cancer. With this structure in place, it will be relatively easy to expand the hospital system to include noncancer specialist services for NCDs, such as, cardiology, diabetes, respiratory medicine, and psychiatry. Therefore, investment in a strong health system that encompasses acute and primary care services is an absolute requirement for any national response for the control of NCDs. We recommend that this change in approach be a priority for member states, particularly in LRCs, to achieve an effective global response to SDG3.4.

## LRCs and Health

In 2013, the global population was estimated to be 7.12 billion persons, consisting of 0.85 billion persons in LICs (11.9%), 2.55 billion Lo-MICs (35.8%), 2.45 billion in upper-middle-income countries (34.4%), and 1.27 billion in high-income countries (HICs; 17.8%).^13^ Although NCDs affect the poorest communities at all levels of society, this article focuses on the 48% of the world population living in LRCs—LICs and Lo-MICs—where poverty is more widespread and health and cancer care is less affordable than in the rest of the world. The predicted overall increase in global cancer incidence between 2010 and 2030 is 51%, and this will be greater in LICs (81%) and Lo-MICs (68%) than in Up-MICs (60%) or HICs^14^ (38%; Table 1).

**Table 1:**
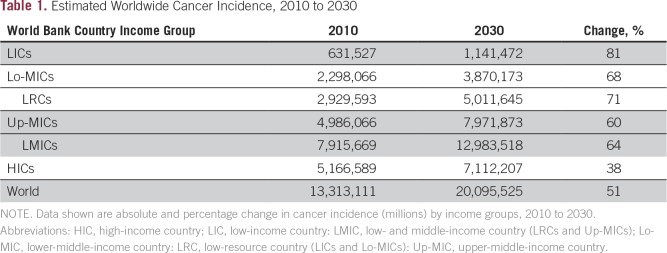
Estimated Worldwide Cancer Incidence, 2010 to 2030

Extreme poverty is the main reason for the current lack of health and cancer services in LRCs, where 40% to 80% of the population survive on less than USD $1.25 per day and more than 50% of the population lives in rural areas. Total spending by LRC governments on health is typically less than 5% of the gross domestic product in LRCs^15^ and averages only $110 per person per year in LICs and $265 in Lo-MICs^16^ (Table 2). Furthermore, patients and families in LRCs must pay out-of-pocket expenses for health and cancer care, and treatment costs frequently result in catastrophic poverty when spending is greater than 30% of personal annual income. Late presentation of locally advanced and incurable disease is frequently a result of the myths and stigma about cancer and reliance on traditional medicine practices mostly given by untrained health workers, which often result in misdiagnosis and ineffective treatment. Widespread poverty plus a lack of roads and public transport make access to the limited cancer treatment facilities impossible for much of the population. The situation is exacerbated by a lack of education about cancer at all levels of society in LRCs, including government bodies, and the commonly held view that cancer is not curable and results in a painful death.

**Table 2:**
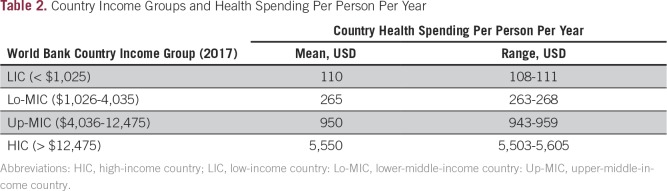
Country Income Groups and Health Spending Per Person Per Year

Surgery^17^ and radiotherapy^18^ have been demonstrated to be cost-effective investments in health and cancer services and to provide major social and economic benefits by preventing unnecessary and premature death and disability. This will have a positive impact on the micro- and macroeconomic environment of a country, improve gross domestic product, and help LRCs to climb out of their parlous financial state that has existed for decades.

The Lancet Commission on Global Surgery has drawn attention to the worldwide inequities and deficiencies in surgery and anesthesia.^19^ Likewise, the Lancet Oncology Commission on Radiotherapy has demonstrated a huge global deficit in radiotherapy, with many LRCs without any radiotherapy at all.^18,20^ The significant deficit in pathology and laboratory medicine services in low- and middle-income countries was documented in a Lancet series,^21^ although the lack of pathology has been noted before.^22-25^ The inadequacy of the surgical workforce,^26-28^ lack of oncology nursing^29^ and palliative care services that include morphine,^30,31^ or the use of falsified or substandard chemotherapy drugs^32-34^ and lack of access to medications in LRCs have been well documented.^35,36^

Health system upgrades are needed at several levels by improving primary health care at health posts and nursing stations in rural and remote areas, level 1 hospitals in district towns and level 2 hospitals in regional cities, and major level 3 tertiary hospitals in capital cities.^37^ Despite the lack of political will and the ability to do so, governments face barriers to improving health care in LRCs that include the cost of buildings, equipment, and technology required; an inability to train medical and paramedical staff and to pay for their subsequent employment; and the cost and reliability of the supply chain for medical consumables. A change in emphasis in the approach to NCDs, particularly in LRCs, is needed so that a strong health system is available and diagnosis, treatment, and management become a priority. Without adequate hospital and community facilities and adequately trained staff, any improvement in the SDG Target 3.4 is unlikely to occur.

## NCSP for LRCs

Although cancer services in most LRCs are likely to be suboptimal, a large variation exists between and within most countries. For example, there is no radiotherapy available in some LRCs and in others the equipment will have long passed its use-by date and may not be suitable for the treatment of patients, even for palliation.^20^

A single-model NCSP will not be applicable for all LRCs as the aim for cancer services will also vary according to the wishes of the particular LRC government. We propose a NCSP with cancer centers developed in a coordinated fashion within the LRC to achieve improved patient access to management, diagnosis, and treatment of cancer. This will be a step-wise process over many years and will require a planning process that involves both public and private hospitals. The aim is to establish a national cancer center (NCC) and a network of several regional cancer centers (RCCs).

We recommend that the NCC be a part of a level 3 hospital in major capital city and equivalent to a comprehensive cancer center in an HIC. It should have the complete range of facilities for the diagnosis, management, and treatment of cancer, including surgery and anesthesia, radiotherapy/clinical oncology chemotherapy/hematology, and palliative care. In addition to updating cancer equipment, the NCC would be a part of a hospital that includes cancer support specialties, such as pathology, blood bank, and diagnostic radiology, at the one site. The NCC would be a focus for education and training of medical, paramedical, and administrative staff, and would also be involved in cancer education and prevention, including vaccination programs plus screening, research, and the establishment of a national cancer registry. Using the hospital as a base would make it easier to expand the noncancer specialties to support other NCDs. RCCs would also have a full range of cancer equipment and technology plus cancer support specialties with the main aim of providing treatment closer to home. These facilities would be in well-developed level 1 and 2 hospitals in regional cities and organized in a hub-and-spoke fashion from the NCC with sharing of staff, subspecialization, common treatment protocols, and a multidisciplinary approach to cancer management.

## PLANNING FOR THE NCSP

Planning for the NCSP would start with an audit of hospital and cancer services within the LRC to determine what is present, what is missing, and what is needed, as well as where the NCC and RCCs should be sited. This would be the most difficult and prolonged period of the NCSP as it would involve discussion and interaction by a multitude of in-country and overseas experts in health, cancer, and finance. The final outcome would undoubtedly be influenced by the existing staff and facilities in the LRC and the funds available for the NCSP project.

In some instances, it may be more realistic to have a series of plans for the phased development of the NCSP, rather than trying to solve the process at once. This will depend on the size of the LRC nation and its population as well as the initial status of health and cancer services. Each LRC will have different requirements for the establishment of the NCSP. Levels of equipment, staffing, and infrastructure of the NCC and RCCs would depend on the finances available and would only be decided after extensive discussions between the national cancer unit (NCU), LRC government, and overseas and local experts.

It is vitally important for the ultimate success of the NCSP to ensure that the LRC government has a strong buy-in, extensive involvement, and a sense of ownership of the project.

Organizations needed for the NCSP:

- An NCU
- A central cancer office (CCO)
- A cancer partnership (CP)
- A cancer fund (CF)

## NCU

An NCU should be within the LRC to lead and direct the NCSP. An NCU should be established within the ministry of health and funded by the CF. The director would ideally be a respected senior medical administrator or cancer specialist. The NCU would be the in-country coordinator of the NCSP and would act as a focal point for the cancer activities of the LRC. Heads of government, members of parliament, the ministry of health, treasury, ministry of finance, ministry of education and training, colleges and universities, professional societies and technical organizations, WHO country office, local embassies, the national cancer society, social society, and other experts would interact via the NCU.

The NCU and LRC government would work in close cooperation with the CCO, CF, and CP to ensure that the NCSP achieves its outcomes in a timely manner. Initially, the NCU, CCO, and CP would undertake the needs assessment within the LRC as outlined above. This would include details of the buildings, infrastructure, equipment, technology, staffing, and training needed. Access to a stable electricity supply, air conditioning, clean drinking water, and contracts for maintenance and spare parts is also needed. The cost of overseas experts to visit the LRC for education and training would also need to be included.

The NCU and CCO would determine an estimate of the total cost and a timeframe for the establishment of the NCC. This would be forwarded to the CCO for review and approval by the grants committee and CCO Board. Funding would be restricted to intervals of 2 years and would be subject to a performance-based assessment so that only effective and successful programs would receive continued funding. The National Cancer Society (NCS) would also work with international banks and other potential funders, such as PricewaterhouseCoopers, KPMG, Rotary International, and the NCU to establish cost-free patient access from outreach clinics to the NCC or RCCs for diagnosis and treatment, and accommodation if needed. The NCU, ministry of health, and NCS should promote cancer education, prevention and early detection measures, screening, a national cancer registry and palliative care facilities. The NCU and CP as well as the ministry of education and training, universities, and colleges would be responsible for the training of professional staff for hospitals and medical specialties as well as an accreditation process for hospitals and staff.

## CCO

The CCO would be required to assist the NCU with appropriate governance, coordination, and operation of the NCSP. The location of the CCO would ideally be in major capital city alongside international government agencies, such as the WHO in Geneva, Switzerland.

Funded by the CF, staff would include a chief executive officer with an elected board, and selected members of the NCU, a CP, a CF, and a grants committee. Establishing the CCO would require a planning committee of 10 to 12 members from health, cancer, and finance who have experience in LRCs and a willingness to support the overall program. For those from health and cancer backgrounds, this would include senior members of the colleges of surgery, medicine, radiation oncology, pathology, radiology, general practice, and palliative care. A number of experts with global health financing experience would be recruited for the CF.

The planning committee would meet to discuss the feasibility of the NCSP and appoint members of the CCO board, CP, and CF. Likely LRCs for pilot studies of the NCSP would also be selected. During the development of the NCSP, the NCU, CCO, CP, CF, and LRC government would be involved and would develop close cooperation to ensure the NCSP meets its commitments and time scales.

## CP

A CP works in the LRC with the NCU to undertake a needs assessment, followed by infrastructure, installation of equipment, and involvement in the education and training of professional staff. The CP would act as an umbrella organization to bring together in-country and overseas experts from relevant professional colleges, universities, and societies who have experience in the relevant aspects of health and cancer services. CP groups would include surgery and anesthesia, radiotherapy/clinical oncology, medicine, hematology/oncology/blood bank, pathology and laboratory medicine, diagnostic radiology, palliative care, pediatric oncology, and general and oncology nursing. Also included would be paramedical staff for each specialty, such as radiographers, laboratory staff, technicians, medical physicists, biomedical engineers, and information technologists.

Interaction with other medical specialty groups in cardiology, diabetes, and respiratory medicine for NCDs would be developed as part of their association with the NCSP. Also included would be the parallel development of psychiatry, pediatrics, obstetrics, and gynecology plus other specialties needed to support the health aspects of the 2030 Agenda. CP groups would play an essential role in the initial needs assessment by the NCU, COO, and CP to determine the requirements of the NCSP. Members from each specialty would be asked to provide an outline of the equipment required for their specialty to operate at basic, intermediate, and advanced levels. A training program would be for current in-country professional staff, including for the assessment of competency. New professional staff would be involved of in an education program at colleges and universities to ensure that continuing in-country training is developed for the future. For each specialty, this would involve extensive discussion and agreement with relevant overseas experts and in-country members of professional societies, colleges, universities, the NCU, and government.

## CF

A CF would develop long-term sustainable funding for the NCSP. Establishment of a CF is critical for the success of the NCSP, as without long-term funding it will not occur. Funds are needed for the NCU, CCO, CF, and CP and for buildings, infrastructure, equipment, consumables and technology, service and maintenance contracts, and for the training and employment of medical and paramedical professional staff, including hospital and government administrative staff.

A global CF has been proposed^38^ and there has been a recent focus on increasing global funding for health.^3,16^ Other methods, such as innovative financing, have also been suggested.^39^ Traditional funding sources, such as multinational and local or regional banks, philanthropic organizations, and official development assistance from overseas HICs, are still potential funding sources. The role of private-public partnerships and exploring partnership governance in global health has recently been examined by the National Academies of Sciences, Engineering, and Medicine.^40-42^ Donations from in-country wealthy citizens or companies would increase the sense of ownership of a NCSP. The Mwanza Cancer Project in Tanzania is an example of local initiative.^43^ Another is the use of money from tobacco taxation that has supported the BP Koirala Cancer Hospital in Nepal since 1994.

The Global Fund to fight AIDS, Tuberculosis and Malaria, or The Global Fund, has been one of the most successful medical fundraising initiatives over the last two decades and has significantly improved diagnosis, treatment, and outcomes for millions of patients with HIV/AIDS^16,44,45^ (Table 3). It provides an excellent model for funding a NCSP in LRCs, and an approach similar to that of The Global Fund would be a suitable working model for health and cancer services in LRCs. Our suggestion to expand the donor base to tackle priority health issues in LRCs using cancer services as a focus is not intended to imply any reduction in funding for HIV/AIDS. There has been significant progress with HIV/AIDs through The Global Fund and more funds are needed. However, it is worth noting that cancer results in more deaths worldwide each year than do HIV/AIDS, tuberculosis, and malaria combined.

**Table 3:**
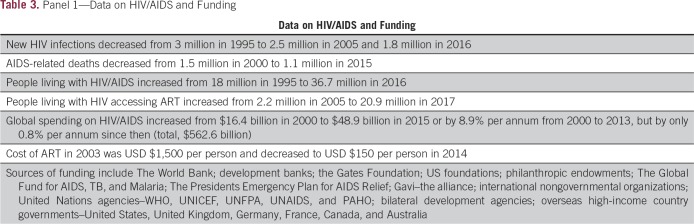
Panel 1—Data on HIV/AIDS and Funding

Funding for global health is increasingly complex and it is essential that the CF group includes members with experience in relevant programs to boost health finances in LRCs. A mechanism to ensure that funding is properly accounted for is also crucial.^46^ Like the HIV/AIDS program, funding by the CF would aim to make the NCSP cost free for patients with cancer as part of developing universal health cover for the nation. Otherwise those who live in poverty will not be able to afford any available treatment.

Because a sense of ownership by the LRC is important for the success of the NCSP, a contribution by the LRC government to staff salaries, for example, would ensure the long-term viability of the program. Although funding is unlikely to be the same for each LRC, there may be a benefit for a particular LRC to pool official development assistance funds from several embassies for the NCSP.

## TRAINING AND EDUCATION OF PROFESSIONAL STAFF

Developing training and education is essential to enable the delivery of safe, accurate, and effective treatments in the above disciplines. In view of the large numbers of different professional staff required, a train-the-trainer practical approach is recommended. Training should take place in-country using the equipment available rather than being done entirely in overseas countries. Training will be needed at several levels for persons who may have worked in health and cancer services in LRCs for many years, but who lack formal training as well as for new recruits into their particular specialties who will require education at an in-country university or college and hands-on practical training.

The composition of the training programs will be decided by the CP and in-country experts in each specialty and will include theoretical and practical training with assessments of competency. Each CP specialty should be expected to provide a complete team of qualified professionals from chosen overseas departments to be responsible for the delivery of the training required for in-country staff in their own specialty at the NCC.

CP groups should be expected to be actively involved in the operation of the new departments from the initial start-up for a period of up to 2 years. This would involve working with in-country staff in a hands-on approach to patient treatment and management to ensure they can work as safe, accurate, and efficient professionals. CP groups would also provide ongoing support after the initial training period via teleconferencing^47-49^ and online support and twinning arrangements.

It is also important that in-country persons are required to undergo continuing professional development after the completion of their initial course of training. The absence of a certificate may make employment overseas more difficult, but the brain drain effect can also be minimized by incentives from the LRC government, such as one that provides hospital housing or wage increases in exchange for undertaking additional training.

## DISCUSSION

The 2030 Agenda is an ambitious plan that attempts to change the world by improving the well-being of all people and ensuring that no one is left behind. The WHO Global Action Plan for NCDs 2013 to 2020 aims for a 25% reduction in premature deaths from NCDs by 2025 (25 by 25) and the 2030 Agenda has proposed a 30% reduction by 2030 (30 by 30). Neither of these goals are likely to be achieved without significant additional funding and a change in approach that involves a commitment to the development of a strong health system, particularly in LRCs.

WHO has introduced Best Buys as a series of interventions for each of the four key risk factors and the four key diseases areas for NCDs. As stated previously, the Best Buys are essentially prevention measures and will take several decades to affect survival; however, a strong health system is an essential requirement for the management of NCDs and other health-related SDG targets.

Unfortunately, the greatest burden of NCDs falls on the poorest countries that are least likely to be equipped or able to afford the control of NCDs. We therefore propose a NCSP that will concentrate on LRCs as a result of the high incidence of premature NCD deaths, widespread poverty, and poorly developed health systems (Table 4). The NCSP will also have a substantial flow-on effect and improve the overall health system that would support the other NCDs and cancer.

**Table 4:**
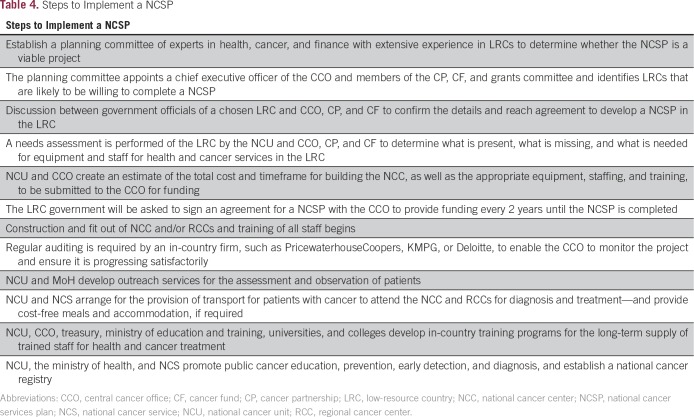
Steps to Implement a NCSP

The social sector also has an important and often undervalued role to play in the delivery of cancer services, particularly in LRCs. As access to care is a major problem in LRCs, this means supporting the social and financial needs of patients and providing access to care during treatment and palliation. In addition, like HIV/AIDS treatment, cancer treatment needs to be cost free to the population so that the uptake is maximized. Although we have not suggested any particular approach to funding, we have drawn attention to the results of HIV/AIDS funding as an example of what can be achieved.

We also suggest that the NCSP should initially be started as a pilot study in one or two LRCs to properly assess its feasibility before expanding to include other LRCs. The NCSP program will require international collaboration from overseas experts in finance, health, and cancer to support the LRC government in leading the development of the NCSP. Success will depend on strategic planning and providing the right balance of overseas support and guidance to ensure that there is in-country ownership and control of the program. The success of HIV/AIDS treatment has provided a proof of concept of what can be achieved and we urge the global cancer community to take immediate action to end the global inequality in cancer care (Table 5).

**Table 5:**
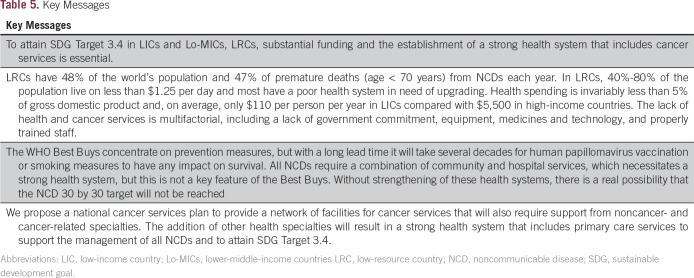
Key Messages
